# The development of SSR markers based on RNA-sequencing and its validation between and within *Carex* L. species

**DOI:** 10.1186/s12870-020-02792-8

**Published:** 2021-01-06

**Authors:** Lingyun Liu, Xifeng Fan, Penghui Tan, Juying Wu, Hui Zhang, Chao Han, Chao Chen, Lulu Xun, Weier Guo, Zhihui Chang, Ke Teng

**Affiliations:** 1 College of Grassland Science, Beijing Forestry University, Beijing, 100083 China; 2 Beijing Research and Development Center for Grass and Environment, Beijing Academy of Agriculture and Forestry Sciences, Beijing, 100097 China; 3 Beijing Chaoyang Foreign Language School, Beijing, 100000 China; 4 Shaanxi Engineering Research Center for Conservation and Utilization of Botanical Resources, Xi’an Botanical Garden of Shaanxi Province (Institute of Botany of Shaanxi Province), Shaanxi, 710000 China; 5 Department of Plant Biology, University of California, Davis, Davis, CA USA

**Keywords:** *Carex* L., Illumina RNA-sequencing, Gene function annotation, SSR marker, Marker polymorphism, Genetic cluster

## Abstract

**Background:**

*Carex* L. is one of the largest genera in the Cyperaceae family and an important vascular plant in the ecosystem. However, the genetic background of *Carex* is complex and the classification is not clear. In order to investigate the gene function annotation of *Carex*, RNA-sequencing analysis was performed. Simple sequence repeats (SSRs) were generated based on the Illumina data and then were utilized to investigate the genetic characteristics of the 79 *Carex* germplasms.

**Results:**

In this study, 36,403 unigenes with a total length of 41,724,615 bp were obtained and annotated based on GO, KOG, KEGG, NR databases. The results provide a theoretical basis for gene function exploration. Out of 8776 SSRs, 96 pairs of primers were randomly selected. One hundred eighty polymorphic bands were amplified with a polymorphism rate of 100% based on 42 pairs of primers with higher polymorphism levels. The average band number was 4.3 per primer, the average distance value was 0.548, and the polymorphic information content was ranged from 0.133 to 0.494. The number of observed alleles (Na), effective alleles (Ne), Nei’s (1973) gene diversity (H), and the Shannon information index (I) were 2.000, 1.376, 0.243, and 0.391, respectively. NJ clustering divided into three groups and the accessions from New Zealand showed a similar genetic attribute and clustered into one group. UPGMA and PCoA analysis also revealed the same result. The analysis of molecular variance (AMOVA) revealed a superior genetic diversity within accessions than between accessions based on geographic origin cluster and NJ cluster. What’s more, the fingerprints of 79 *Carex* species are established in this study. Different combinations of primer pairs can be used to identify multiple *Carex* at one time, which overcomes the difficulties of traditional identification methods.

**Conclusions:**

The transcriptomic analysis shed new light on the function categories from the annotated genes and will facilitate future gene functional studies. The genetic characteristics analysis indicated that gene flow was extensive among 79 *Carex* species. These markers can be used to investigate the evolutionary history of *Carex* and related species, as well as to serve as a guide in future breeding projects.

**Supplementary Information:**

The online version contains supplementary material available at 10.1186/s12870-020-02792-8.

## Background

The genus *Carex* L. belongs to the Cyperaceae family and is an enormous genus. It is one of the most vital genera of vascular plants in the environment [1], with more than 2000 species widespread all over the world [2] and nearly 500 species in China [3]. *Carex* species are widely used as a ground cover for home lawns as well as for slope stabilization in many parts of the world because of cold and drought tolerance [4], trample resistance, and high ornamental value [5, 6].

Previous studies focused on geographical distribution, phylogeography, and origin area of *Carex*. Benítez-Benítez et al. [7] found obvious genetic differentiation between two *Carex* sister species in the western Mediterranean, and pointed out that geographic barriers played dominant role in restricting gene flow. Míguez et al. [8] revealed late Miocene-Pliocene aridification of the Mediterranean shaped the phylogeography of *Carex* sect. *Rhynchocystis*. Martín-Bravo et al. [9] proved that *Carex* originated in the late Eocene in East Asia which has a productive diversity of *Carex*. Previous studies clarified the phylogenetic structure of *Carex* into at least four major clades, including *Siderostictae* clade, core *Carex*, *Vignea*, and *Caricoid* clade [10]. However, a supermatrix analysis combining ETS, ITS and *mat*K DNA regions indicated that over-reliance on morphological characters was inappropriate for the delimitation of natural groups [11].

Molecular markers are powerful tools for genetic diversity analysis which is the basis of accelerating plant breeding process. Currently, commonly used molecular markers mainly include ISSR, RAPD, RFLP, AFLP, SSR, and SRAP [12]. Due to the advantages of abundant, multi-allelic, highly polymorphic and codominant, simple repeat sequence (SSRs) for genetic research are a good choice to reveal the mechanism of genetic genes in plants [13, 14]. RNA-sequencing is an effective tool to obtain SSRs with higher rate of transferability for non-sequenced genomes and non-model organisms [15, 16]. It has been demonstrated that SSRs obtained from one species could be used to detect diversity in related species and even in other genera of the same family [17, 18]. SSR has been widely used in genetic mapping, relationship studies [19], cultivar identification [20, 21] and analysis of plant genetic diversity [22]. Hitherto SSR markers have been widely applied to plant research, such as *Zea mays* [23], *Citrullus lanatus* [24], *Triticum aestivum* and the *genus Cerasus* species [25].

In previous studies, there have been many molecular marker studies on *Carex*, which also included SSR method. M’Baya et al. [26] utilized 14 SSRs isolated in *Carex kobomugi* to test genetic structure of *Carex hebes* and *Carex breviculmis* and possessed a high level of genetic variation. Meanwhile, other useful molecular markers also used to investigate the diversity between *Carex* species. Liu et al. [3] reported 30 SSR markers from *C. moorcroftii*, which provide an available tool to explore the genetic structure and phylogenetic evolution. Starr et al. [27] found that *mat*K barcoding could distinguish between 47% of *Carex* materials and clearly distinguish phylogenetic diversity relationships in NJ evolutionary trees. Ning et al. [28] proved that ISSR molecular markers are a powerful tool for studying the genetic diversity of *Carex*. They all found a result that the diversity between *Carex* species were complex. Nagasawa et al. [29] found that the evolutionary relationships of *Carex* populations could result in a low level of polymorphism in the populations. Man et al. [30] compared genetic variation and population structure of 15 *C.breviculmis* populations in Korea and indicated that gene flow was extensive. Although certain progress has been made in studying *Carex* genetics using molecular markers, there are few studies on SSR molecular markers based on Illumina RNA-sequencing. And the number of materials used are less in the previous research. Moreover, compared to the studies of crops and model plants, molecular studies of *Carex* are still lacking. It is of great economic value to research the relationships and diversity among *Carex* species at present.

In our previous study, we used the single-molecule long read sequencing method to investigate the transcriptional regulating network of *Carex breviculmis* in response to shade tolerance [31]. In the present study, we further explored the SSRs based on the previous Illumina sequencing dataset. The aims of this study were: (1) to enrich *Carex* transcriptome information and get a better understanding of the function categories from the annotated genes, (2) to develop SSR markers and validate their polymorphism levels, (3) to investigate the genetic background between *Carex* germplasms.

## Results

### Illumina sequencing and de novo assembly of transcriptome

The transcriptome of *Carex breviculmis* was sequenced using the HiSeq^TM^ 2000 platform. A total of 43.67Gb clean data was obtained. The clean data of each sample reached 6.32Gb, and the Q30 base percentage was above 94.03%. A total of 36,403 unigenes were assembled, of which there were 12,657 unigenes with a length of more than 1 kb. The N50 of the unigene was 2016, indicating a high assembly integrity.

### Gene annotation based on different databases

**Fig. 1:**
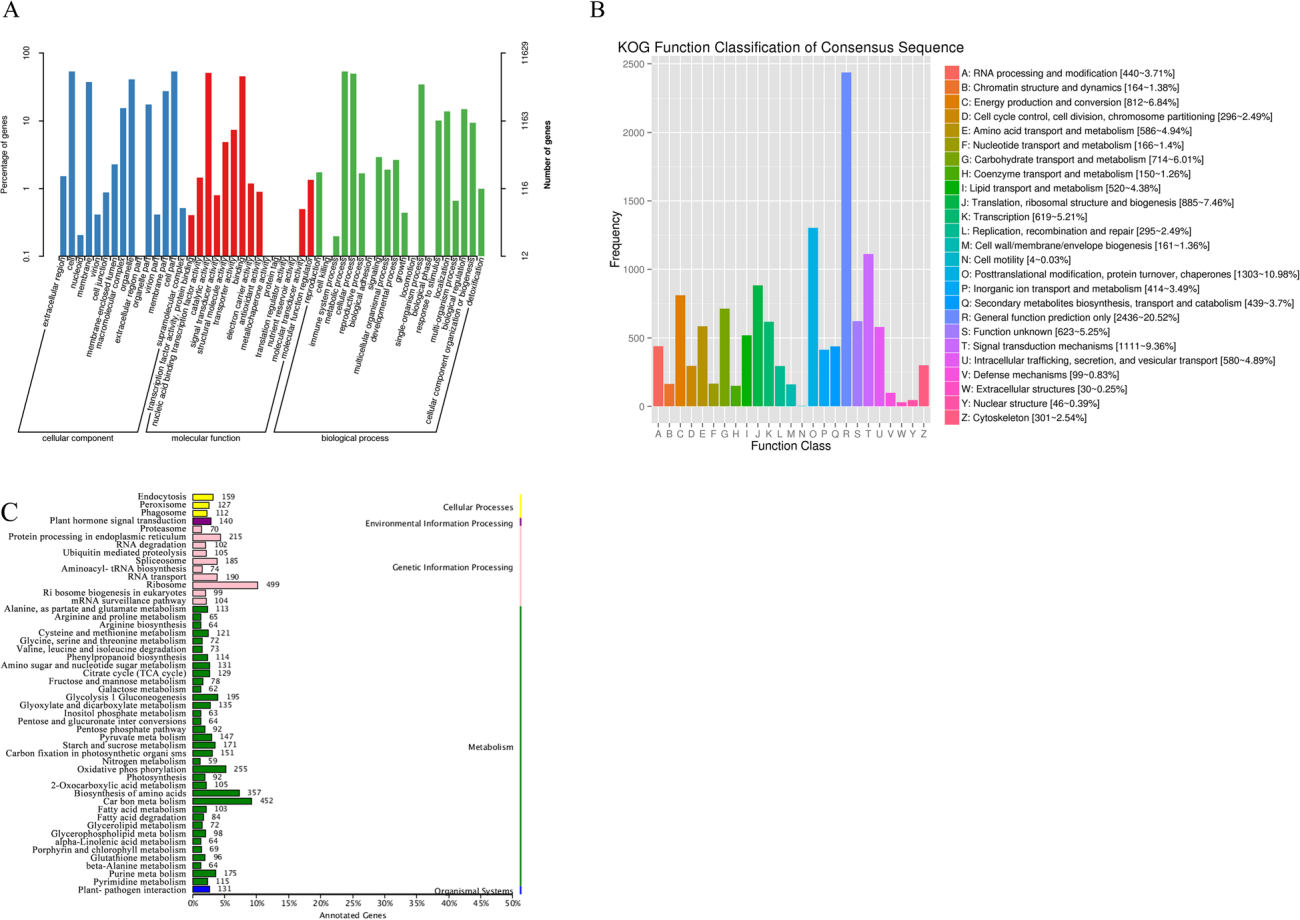
The gene annotation results based on GO, KOG and KEGG databases. **a** Gene Ontology (GO) annotated graph of *Carex*. The results showed that 11,629 unigenes were divided into three main GO categories: ‘Cellular process’, ‘Metabolic process’, and ‘Single-organism process’. **b** EuKaryotic Ortholog Groups (KOG) classification of *Carex*. Eleven thousand eight hundred seventy-one unigenes were classified into 25 functional categories. **c** Kyoto Encyclopedia of Gene and Genomes (KEGG) classification of *Carex.* The X-axis and Y-axis represent the number of genes and the number of subgroups in each metabolic pathway respectively

Based on homology analysis of the sequence, 11,629 unigenes (31.95%) were divided into three main GO categories and 50 sub-categories. The GO classification includes ‘Cellular process’, ‘Metabolic process’, and ‘Single-organism process’. (Fig. 1a). The ‘Cell’ was the largest subgroup of cellular components group. The next largest group was ‘Cell part,’ followed by ‘Organelle’, ‘Nucleoid’ and ‘Macromolecular complex’. The categories ‘Catalytic activity’ and ‘Binding’ among ten different molecular function categories for the *Carex* unigenes were also abundant. According to the KOG database, 11,871 unigenes were categorized into 25 functional groups and 20.52% of unigenes were annotated to ‘General function’ cluster. ‘Post-translational modification’, ‘Protein turnover’ and ‘Chaperones’ (1303 unigenes, 10.98%) was the next largest group and followed by ‘Signal transduction mechanisms’ (1111 unigenes, 9.36%). Alternatively, ‘Nuclear structure’ (46 unigenes, 0.39%), ‘Extracellular structures’ (30 unigenes, 0.25%). Unigenes in ‘Cell motility’ (4 unigenes, 0.03%) groups were significantly less than the above three groups (Fig. 1b). Based on the KEGG database, a total of 8440 unigenes were found, including 40 biological pathways belonging to five large groups (Cellular Processes, Genetic Information Processing, Environmental Information Processing, Metabolism, and Organismal Systems). Three main pathways included Ribosome (499, 5.91%), Carbohydrate metabolism (452, 5.36%), and Biosynthesis of amino acids (357, 4.23%) were in these 50 pathways (Fig. 1c).

**Fig. 2:**
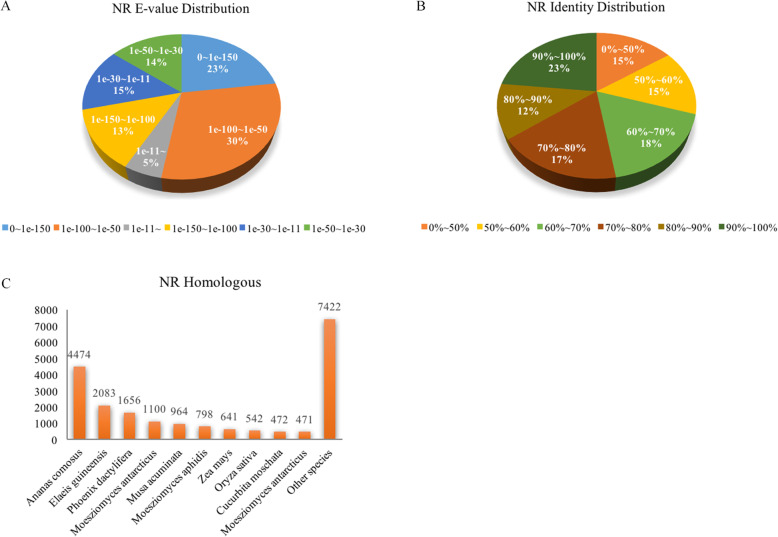
Homology searches of *Carex breviculmis* unigene and characteristics of non-redundant protein databases (Nr). **a** The E-value distribution of unigene BLASTx hits for every assembly. **b** BLASTx hit profiles for every assembly of unigene. **c** Distribution of acessions hit by BLASTx at the top of each assembly of unigene

Based on the database of NCBI non-redundant nucleotide, the E-value distribution revealed that 23.00% of unigenes yielded significant hits (Fig. 2a), and approximately 35.00% of unigenes exhibited greater than 80% identity (Fig. 2b). NR protein sequences alignment results revealed that 21.69% could be aligned with *Ananas comsous*, 10.10% could be aligned with *Elaeis guineensis*, and 8.03% could be aligned with *Phoenix dactylifera* (Fig. 2c).

### Frequency and distribution of SSRs in the transcriptome

**Fig. 3:**
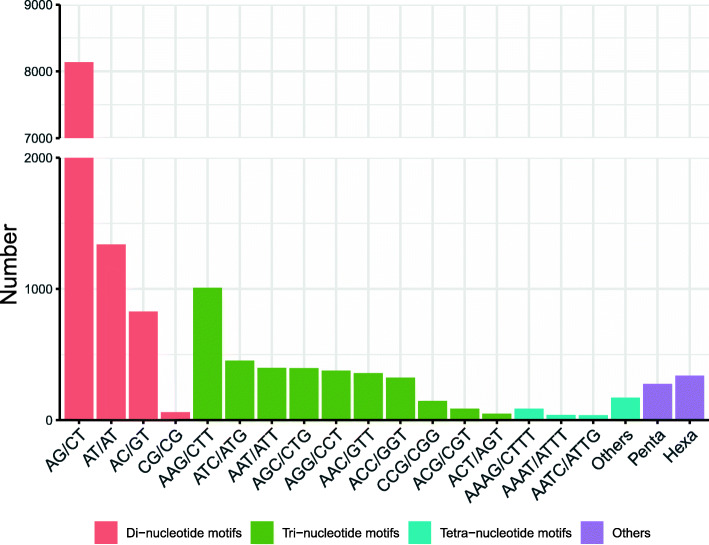
Type distribution of SSRs identifed in *Carex breviculmis* unigenes

A total of 36,403 unigenes were scanned using the MISA software and 8776 SSR loci were detected (Table 1). The SSR locus in the transcriptome has six types and the number of each repeat type varies greatly. The single repeat motif accounting for 64.93% ranked the most abundant type, whereas the hexa-nucleotides accounting for 1.11% was the least abundant type. The most abundant Di-nucleotide repeats were AC/GT (8138; 18.02%) and followed by AT/AT (1339; 3.10%), AC/GT (827; 1.83%). The most plenty Tri-nucleotide repeats were AAG/CTT (1008; 2.23%) and followed by ATC/ATG (454; 1.01%). Meanwhile, the most affluent tetra-repeat motif types were AAAG/CTTT (87; 0.21%). The number of hexa- and penta-nucleotide motifs were 399 (0.88%) and 377 (0.83%), respectively (Fig. 3).

**Table 1:** Prediction of SSRs out of our transcript datasets of *Carex breviculmis*

Item	Number
Total number of sequences examined	36,403
Total size of examined sequences (bp)	41,724,615
Total number of identified SSRs	8776
Number of SSR containing sequences	6018
Number of sequences containing more than one SSR	504
Number of SSRs present in compound formation	20
Mono nucleotide	5699
Di nucleotide	1873
Tri nucleotide	582
Tetra nucleotide	63
Penta nucleotide	15
Hexa nucleotide	20

### Development and transferability assessment of novel SSRs

We designed and synthesized 96 pairs to amplify 11 phenotypic difference *Carex* materials (Table S1). Among them, 42 (43.75%) pairs can amplify several bands and have high polymorphism. The primers indicated good transferability between different *Carex* species. The number of not amplified bands accounts for 15.6% and others showed low polymorphism or no polymorphism.

### Genetic diversity statistics

In the set of 42 SSRs, we recognized 180 marker alleles across the 79 accessions. Among the 42 SSRs, PIC value ranged from 0.133 and 0.494, with an average of 0.259. SSRs displayed wide genetic variation among accessions. The genetic diversity between *Carex* materials was investigated by cluster analysis, principal component analysis. The polymorphic ratio was 100% and an average of 4.3 primers was amplified per primer. The number of observed alleles (Na), the number of effective alleles (Ne), Nei’s (1973) gene diversity (H), and the Shannon information index (I) were 2.000, 1.376, 0.243, and 0.391, respectively, indicating that the genetic diversity between the *Carex* accessions was high. We also calculated the genetic distance between accessions (Table S2), which ranged from 0.222 to 1.000. The genetic distance average value was 0.548.

### Cluster analysis of *Carex* based on SSR markers

In order to reveal the classification information of *Carex* species, we obtained the allele frequency according to our original data. Instead of using a priori classification such as provenance or taxonomy, we used NJ, PCoA and UPGMA cluster analysis and combined the results to explore the genetic information and classification of all accessions.

**Fig. 4:**
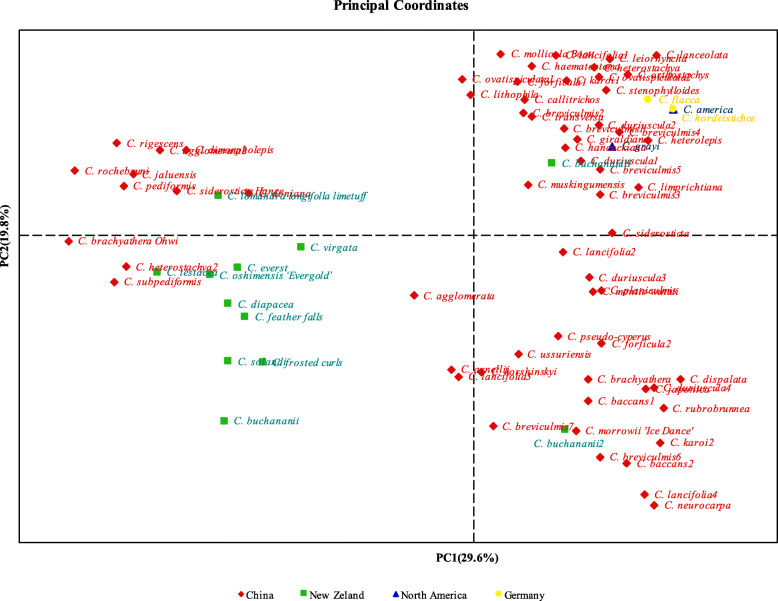
PCoA analysis of 79 materials of *Carex.* The first principal component accounts for 29.6% and the second principal component accounts for 19.8%. The red diamond represents the germplasm resources from China, the green square represents New Zealand, the blue triangle represents North America, and the yellow circle represents Germany

Principal coordinate (PCoA) results showed that Axis 2 separated and generated two genetically differentiated groups of *Carex* accessions (Fig. 4). The first principal component accounts for 29.6% and the second principal component accounts for 19.8%.

**Fig. 5:**
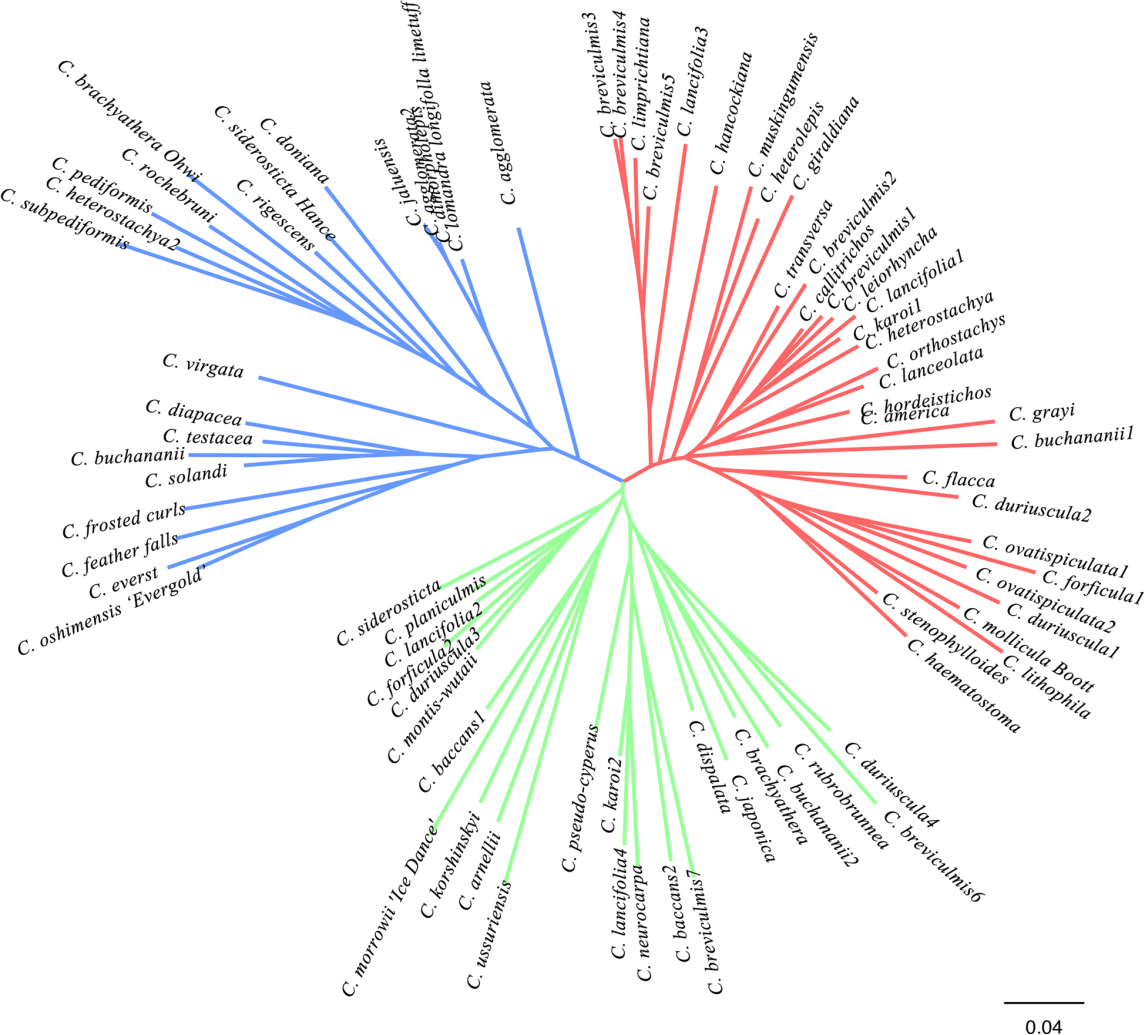
N-J phylogenetic tree of 79 *Carex* accessions. N-J tree included three major clusters, including Group I (22 accessions) which is blue, II (24 accessions) green and III (33 accessions) red

Based on the distance calculation method of Shered Allele, the Neibor-Joining phylogenetic analysis divided 79 *Carex* accessions into three groups (Fig. 5). The Group I has a total of 22 materials and *C. jaluensis*, *C. dimorpholepis*, *C. agglomerata2* are grouped into very similar categories. Also the commercial plant materials from New Zealand are grouped into one category in Group I, including *C. virgata*, *C. frosted curls*, *C. solandi*, *C. oshimensis’Evergold’*, *C. feather falls*, *C. buchananii*, *C. everst*, *C. diapacea*, *C. testacea*, *C. lomandra longifolla limetuff*. The Group II includes 24 materials, *C. subpediformis* and *C. jaluensis* are the most similar accessions. The Group III includes 33 materials and most of which are from all over China, but also four materials are from Germany and North America. It is worth mentioning that the *C. breviculmis* collected from various provinces in China are all classified into this group.

**Fig. 6:**
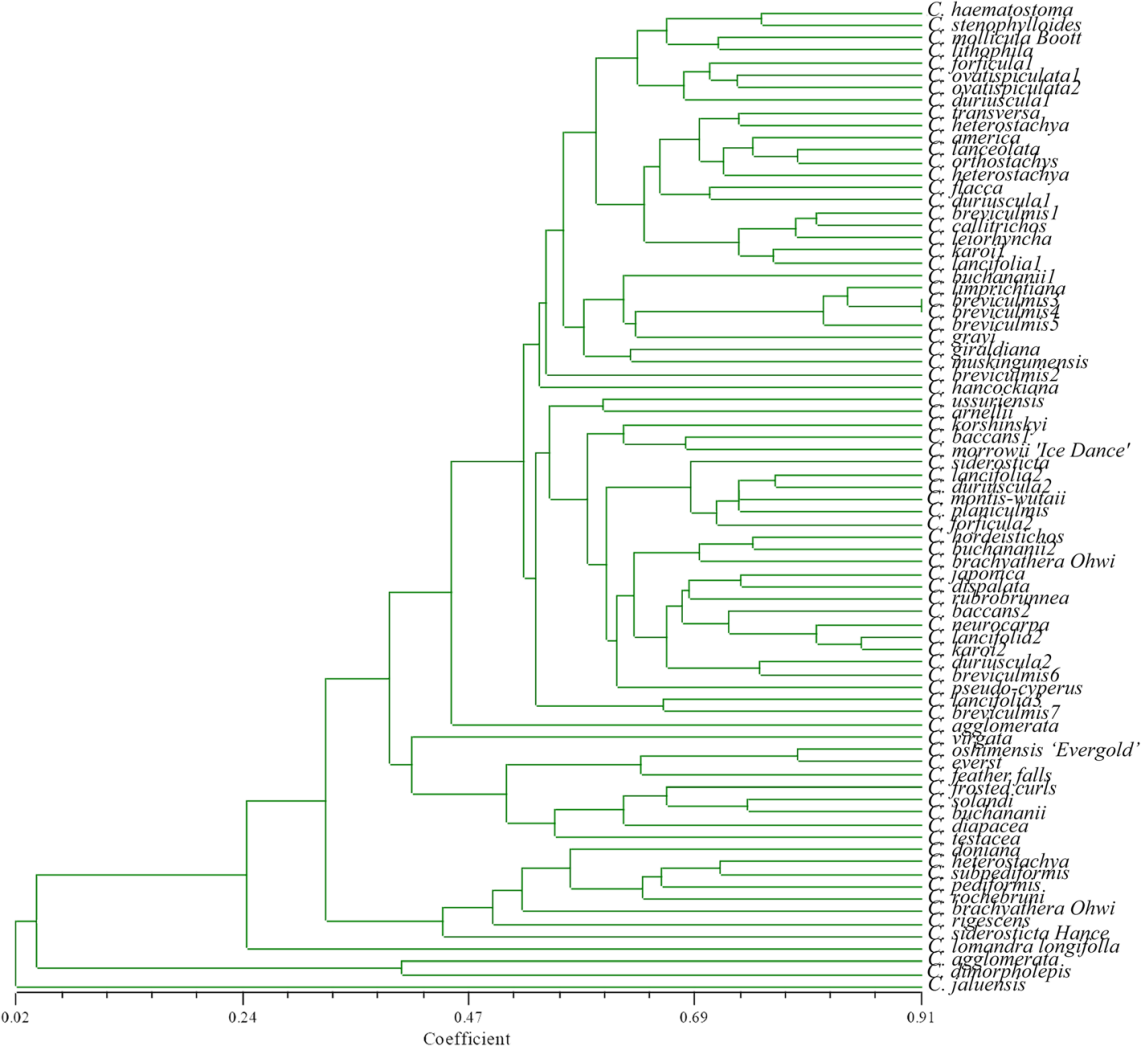
UPGMA clustering analysis of 79 *Carex*

Based on the SSR original data, Dice coefficient method was used to calculate the similarity. A total of 79 materials were clustered using the UPGMA method (Fig. 6). The similarity between genotypes was 0.070 ~ 0.786. Through UPGMA clustering, 79 accessions of germplasm resources were divided into two major groups. *C. jaluensis* was assigned to a separate group. The remaining 78 domains of *Carex* accessions were divided into two subgroups, and *C. agglomerata*, *C. dimorpholepis* and *C. lomandra longifolla limetuff* were classified into one group. While nine accessions from New Zealand were clustered into one group when the genetic distance was 0.42, but *C. lomandra longifolla limetuff* was not clustered into this group. Although it was not clustered with the accessions from New Zealand, the genetic similarity was relatively high. Regarding the collection sources of *Carex*, there is a certain degree of gene exchange between *Carex* germplasm from China and New Zealand, Germany and North America. However, the resources cannot be divided completely by region in general. The Sorensen-Dice correlation coefficient was *r* = 0.941, indicating that the clustering results were reliable.

### AMOVA analysis of *Carex* accessions based on different methods

In order to evaluate the genetic differences between these germplasms, we calculated the F_ST_ values between all pairs of accessions for the two levels studied (origin and genetic classification). Through different analysis of source, the AMOVA indicated that 88% of the total genetic variation of 79 accessions was within populations, and 12% was among populations. The results of AMOVA analysis showed that some subspecies have large variations within countries, which was consistent with the above results of accessions from different countries. While the results of AMOVA analysis based on NJ cluster analysis showed that 89% within populations (Table 2). F_ST_ values and probability P (rand ≥ data) were 0.117 and 0.052 respectively.

**Table 2:** AMOVA analysis of *Carex* accessions

	Source	df	SS	MS	Est. Var.	%
Regions	Among Pops	3	181.719	60.573	3.667	12%
	Within Pops	75	2082.687	27.769	27.769	88%
	Total	78	2264.405		31.436	100%
Taxa	Among Pops	2	217.629	108.814	3.162	11%
	Within Pops	76	2046.777	26.931	26.931	89%
	Total	78	2264.405		30.093	100%

Note: AMOVA results for 4 regions of origin and 3 taxa. Fst values and probability P (ran≥data) were as follows: Region of Origin (0.117;0.052), Taxa (0.105;0.001)

*Df* Degrees of Freedom, *SS* Sum of squares, *MS* Mean squares, *Est. Var.* estimated variance, *%* proportion of genetic variability

### Fingerprint

Results showed that 42 pairs of SSR markers can be efficient in differentiating between 79 accessions, and a fingerprint map, which can distinguish more varieties at a time, was established (Table S3).

## Discussion

Our previous work reported the transcriptional regulation network of *C. breviculmis* in response to shade stress using single-molecule long-read sequencing [31]. In the present study, we utilized the Illumina RNA-seq data in order to generate SSR markers and thus to explore the genetic diversity of other 79 *Carex* accessions. We released a large quantity of expressed gene sequences (36,403 unigene sequences). In order to get a better understanding of the function categories of *Carex* genes, we searched the GO, KOG, KEGG pathway mapping databases. A total of 20,948 unigenes annotation results were obtained. The results showed that 57.54% of the genes were successfully annotated, reflecting the high transcriptome diversity of *Carex*. Gene Ontology is a global classification system of the gene function, classifies the characteristics of genes into groups of ‘biological processes’, ‘cellular components’ and ‘molecular functions’ [32]. KOG analysis was devoted to classify orthologous groups for eukaryotic complete genomes. KEGG analysis is one of the most frequently used method to analyze gene metabolic products and its putative functions [33]. As in the results of Li et al. [34], the GO database classifies annotation information into three categories: biological processes, cellular components, and molecular functions. The largest subcategory was ‘binding’, while followed by ‘catalytic activity’. We also found that the KEGG pathway had the largest group of genes belonging to the metabolism category, while the genetic information processing category was the second largest group. Based on NR alignment results, 21.69% of sequences could be aligned with those of *Ananas comsous*. It was indicated that *C. breviculmis* and Bromeliaceae family members has closest relationship with respect to protein alignment. *Ananas comsous* belongs to Bromeliaceae family, while *C. breviculmis* belongs to Cyperaceae. Considering the taxonomy of plants, *A. comsous* and *C. breviculmis* seem to be genetically distant species. The reason for the unexpected protein alignment could be the lack of data on Cyperaceae-related species in the current NR database. The results show that it is necessary and urgent to update the genetic database of this genus.

Gene structure analysis was performed based on the unigene library, in which SSR analysis obtained a total of 8776 SSR markers. Among the detected sites, 5699 single nucleotide repeats accounting for the total number of SSR sites of 64.93%. Followed by trinucleotides and dinucleotides (1873 and 582, respectively, 21.37 and 6.63%), which are different from the results that three Nucleotide repetitive sequences are the most abundant repetitive units in radish, with a frequency of 52% [35]. It indicated that the specificity of SSR sites is different in plants. Among all the repeat motifs of 303 functional primer pairs, AC/GT (18.02%) is the most abundant dinucleotide repeat in *Carex breviculmis*.

Although there are a lot of researches on *Carex*, more researches are on a certain kind or a restricted property of it. However, the genetic background and genetic relationship are unclear, especially between and among *Carex* species. It restricted the introduction of *Carex* resources, rational selection of hybrid parents, and genetic engineering breeding. In particular, studies on the genetic level of *Carex* have not revealed evolutionary issues such as genetic relationship.

In this research, we found that 42 SSR primer pairs amplified 178 alleles in 79 *Carex* accessions, with an average of 4.3 alleles per microsatellite. Compared with previous studies, the ratio of polymorphic 100% were higher than the study of *C. sempervirens* used RAPD markers [36]. It indicted that the development efficiency and polymorphism of SSR markers developed by transcriptome sequencing are more efficient than RAPD markers. We also found that PIC value in this research was lower than the value of 0.83 reported by Ning et al. [28] and similar to the value obtained by Nagasawa et al. [29]. PIC values are used to measure the level of population polymorphism in other plants and they depend on the accessions tested. Locus polymorphism can be divided into high level (PIC > 0.5), medium level (0.5 > PIC > 0.25) and low level (PIC < 0.25) according to their information content [37]. In case of the number of *Carex* and molecular markers used in Ning’s study were small, and all of the *Carex* accessions were from the same region in the Shandong Province. Secondly, ISSR used by Ning et al. [28] is probably more efficient than using specie specific SSR markers. The PIC value is similar to Nagasawa et al. [29] research results that 20 of EST-SSR markers developed with low polymorphism in *C. angustisquama* population and King et al. [38] results that identified 11 microsatellite loci from *Carex macrocephala*. Due to the species’ population dynamics, the low genetic polymorphism were obtained, rather than to null alleles at the developed markers. *Carex* germplasms are considered to have the lower PIC values which identified in this study appeared to reflect this low diversity and the low genetic variation in *Carex* resulted from the species history, and not from the characteristics of the used markers.

In cluster analysis, we used a combination of NJ, UPGMA and PCoA analysis methods. Firstly, by the reason of the growth environment is similar and the nine accessions are all commercial materials introduced and domesticated in New Zealand, the germplasm from New Zealand are consistent in the classification to one group. Same with the research of Ning et al. [28], the results of the species from the same area could be classified into one category, which is correlated with geographical distribution and environmental conditions. Secondly, because of similar genetic backgrounds and morphological characteristics or their common geographic origin, *C. grayi* and *C. hordeisticho* of NJ Group III had obvious characteristics. Also *C. breviculmis* and *C. lancifolia* from China were clustered into Group III of closely related species, which is consistent with the traditional classification method. The morphology of these two *Carex* species are very similar, and even hard to distinguish, but it can be achieved using the clustering method of molecular markers.

There was a gene exchange with the Chinese test materials during the cultivation process that *C. buchananii1* and *C. buchananii2* are mixed with Chinese materials to varying degrees. The materials from Germany and North America could not be classified into a single category due to the sample size, but we found that the collection place of the materials would have a certain impact on the classification of *Carex.* Furthermore, through the analysis of principal components, it can be seen that *Carex* plants are not clearly classified. Perhaps they still have large genetic differences among plants of the same genus and no definite result. Therefore, further studies involving more foreign germplasms are still needed in order to better interpret this phenomenon.

In addition, the PCoA based on these genotypic data clearly showed that there was an obvious genetic differentiation among *Carex* accessions. The clustering results of PCoA and UPGMA were partially consistent and showed significant differences among all the analyzed accessions. The result of fingerprint is valuable for *Carex* species with extremely similar appearance that don’t be easily distinguished. Previous studies have showed that SSR markers can be used for plant species diversity analysis and fingerprint development [39]. For example, they were used in establishing the fingerprint of 36 Chinese jujube cultivars with 12 pairs of newly developed SSR primers [40], and eight SSR loci were further recommended as a core marker set for fingerprinting of the tea plant [41]. In this study, we concluded that different combinations of primer pairs could be used to distinguish different species of *Carex*, which is of great significance in the selection of hybrids for breeding new varieties.

## Conclusion

The transcriptomic analysis shed new light on the function categories from the annotated genes and will facilitate future gene functional studies. The genetic background analysis indicated that gene flow was extensive among 79 *Carex* species. These markers can be used to assess genetic diversity and to investigate the evolutionary history of *Carex* and related species, as well as to serve as a guide in future breeding projects.

## Methods

### Plant material and DNA extraction

A total of 79 *Carex* accessions were collected from different locations, including 64 from China, 11 from New Zealand, 2 from the North America, and 2 from Germany. The 64 accessions from China were sampled from the Beijing Botanical Garden, Mount Tai, Xi’an Botanical Garden, Hebei, Shanghai, and the base of the Beijing Academy of Agricultural and Forestry Sciences (Table 3). We have acquired a permission to purchase or collect all of the plant materials under the guidelines of local organizations. The formal identification of the samples was carried out by two plant taxonomists of our group, Dr. Chen and Dr. Xun, referring to the Flora Reipublicae Popularis Sinicae and based on the commercial introductions. Genomic DNA was extracted from healthy young shoots by mixing 10 individual plants of each culture separately using the CTAB method [42]. Using 1% agarose gel electrophoresis to examine the quality and integrity of genomic DNA. DNA concentration was determined through with the NanoDrop 2000 spectrophotometer (NanoDrop, Thermo Fisher Scientific, Wilmington, DE, USA). The extracted DNA was diluted with ddH_2_O to 20 ng/mL and used as the template and inventory for PCR amplification. The extracted DNA was stored at -20 °C [43].

**Table 3:** The 79 *Carex* materials information

Code	Name	Origin
1	*C. haematostoma*	Yunnan, China
2	*C. mollicula Boott*	Beijing, China
3	*C. transversa*	Beijing, China
4	*C. forficula1*	Beijing, China
5	*C. breviculmis1*	Beijing, China
6	*C. breviculmis2*	Beijing, China
7	*C. ovatispiculata1*	Beijing, China
8	*C. leiorhyncha*	Beijing, China
9	*C. lithophila*	Beijing, China
10	*C. ovatispiculata2*	Beijing, China
11	*C. heterostachya*	Beijing, China
12	*C. karoi1*	Beijing, China
13	*C. callitrichos*	Beijing, China
14	*C. lancifolia1*	Beijing, China
15	*C. limprichtiana*	Beijing, China
16	*C. duriuscula1*	Beijing, China
17	*C. breviculmis3*	Beijing, China
18	*C. breviculmis4*	Beijing, China
19	*C. stenophylloides*	Beijing, China
20	*C. breviculmis5*	Beijing, China
21	*C. duriuscula2*	Beijing, China
22	*C. giraldiana*	Beijing, China
23	*C. muskingumensis*	Beijing, China
24	*C. heterolepis*	Beijing, China
25	*C. lanceolata*	Beijing, China
26	*C. orthostachys*	Beijing, China
27	*C. hancockiana*	Beijing, China
28	*C. siderosticta*	Beijing, China
29	*C. lancifolia2*	Beijing, China
30	*C. ussuriensis*	Beijing, China
31	*C. planiculmis*	Beijing, China
32	*C. arnellii*	Beijing, China
33	*C. lancifolia3*	Shandong, China
34	*C. japonica*	Beijing, China
35	*C. montis-wutaii*	Hebei, China
36	*C. korshinskyi*	Xi’an, China
37	*C. forficula2*	Shandong, China
38	*C. duriuscula3*	Beijing, China
39	*C. baccans1*	Xi’an, China
40	*C. dispalata*	Xi’an, China
41	*C. brachyathera*	Xi’an, China
42	*C. agglomerata*	Xi’an, China
43	*C. duriuscula4*	Xi’an, China
44	*C. rubrobrunnea*	Xi’an, China
45	*C. morrowii ‘Ice Dance’*	Xi’an, China
46	*C. breviculmis6*	Xi’an, China
47	*C. baccans2*	Beijing, China
48	*C. neurocarpa*	Shaanxi, China
49	*C. breviculmis7*	Shandong, China
50	*C. lancifolia4*	Shandong, China
51	*C. pseudo-cyperus*	Shandong, China
52	*C. karoi2*	Xi’an, China
53	*C. agglomerata2*	Beijing, China
54	*C. doniana*	Xi’an, China
55	*C. heterostachya2*	Xi’an, China
56	*C. rochebruni*	Xi’an, China
57	*C. dimorpholepis*	Xi’an, China
58	*C. pediformis*	Beijing, China
59	*C. brachyathera Ohwi*	Beijing, China
60	*C. rigescens*	Beijing, China
61	*C. subpediformis*	Shanghai, China
62	*C. jaluensis*	Shanghai, China
63	*C. siderosticta Hance*	Shanghai, China
64	*C. buchananii1*	Shanghai, China
65	*C. buchananii2*	ODERINGS, New Zealand
66	*C. virgata*	ZELANDIA, New Zealand
67	*C. frosted curls*	ODERINGS, New Zealand
68	*C. solandi*	ZELANDIA, New Zealand
69	*C. oshimensis ‘Evergold’*	ZELANDIA, New Zealand
70	*C. feather falls*	ZELANDIA, New Zealand
71	*C. buchananii*	ODERINGS, New Zealand
72	*C. everst*	ODERINGS, New Zealand
73	*C. diapacea*	ODERINGS, New Zealand
74	*C. testacea*	ODERINGS, New Zealand
75	*C. lomandra longifolla limetuff*	ODERINGS, New Zealand
76	*C. grayi*	North America
77	*C. america*	North America
78	*C. flacca*	Germany
79	*C. hordeistichos*	Germany

### Transcriptome sequencing, de novo assembly and function annotation

Transcriptome sequencing of *Carex breviculmis* was performed by Biomarker Technologies for RNA extraction, and sequencing was performed on the HiSeq™ 2000 platform [31]. In this study, TRINITY Version 2.5.1 was used to detect contigs from the same transcript, to determine the distance between contigs and connect them together to obtain contigs with inextensible ends [44]. TGICL software was used to splice these single genes and remove their redundancy to obtain non-redundant single genes. Using the BLAST (http://blast.ncbi.nlm.nih.gov/Blast.cgi) searches (e-value <1E-5) of the assembled unigenes [45, 46]. The sequence is compared to the following databases: Gene Ontology (GO), Eukaryotic Orthologous Groups of proteins (KOG), the Kyoto Encyclopedia of Genes and Genomes (KEGG), and National Center for Biotechnology Information for non-redundant proteins (NR).

SSRs of the transcriptome were identified using MISA [47]. Using Primer3 (http://primer3.sourceforge.net/releases.php) for each SSR primer design. SSR loci contained motifs of two to six nucleotides in size were preferentially selected. The other principle of selection was that six minimum repeating units of di-nucleotide, five tri-nucleotides, and four of all higher order motifes, including tetra-nucleotide, penta-nucleotide, and hexa-nucleotide.

### Identification of SSRs, and primer design

Amplification of SSR markers was carried out using DNA of 11 *Carex* species with large phenotypic differences. Ninety-six SSR primers, which were identified in *Carex breviculmis* using RNA-seq, were randomly selected for this research. All primers were synthesized by RuiBo Biotech. Among the ninty-six synthesized pairs of primers, 42 pairs had higher levels of polymorphisms and were used in further experimental research (Table 4). All reactions were conducted using BIO-RAD T100 Thermal Cycler™. The PCR reaction system were carried out in a total volume of 10 μL, including 5 μL of 2 × Taq Master Mix, 0.2 μL of primer, 2 μL of genomic DNA, and 2.6 μL of ddH_2_O. The thermal profile used for amplifications consisted of 10 min of initial denaturation at 94 °C, followed by 34 cycles of 30 s at 94 °C, 30 s at the optimized annealing temperature, 60 s of extension at 72 °C, and a final extension of 5 min at 72 °C. Used 8.0% non-denatured polyacrylamide gels with a 100-bp ladder marker (TRANSGEN BIOTECH, Beijing, China) to separate successful PCR products and visualized by silver staining [48]. DNA of 79 *Carex* individuals was amplified using SSR primers to analyze genetic diversity. Clear bands on the gel images under the light lamp were observed, with or without bands as (1) or (0).

**Table 4:** The SSRs primer information identified for validation in this study

Primer	#Gene_ID	SSR	Forward primer(5′-3′)	Reverse primer(5′-3′)
CAREX001	F01.1	(AG)6	CACTGGAGAACCTAGCGACC	TTGTACAAGGTCCAGGGAGAA
CAREX006	F01.141	(TC)7	CGTTCCCCGTTTTCTTCTCT	GCCGTCTTCTTTGAAAACCA
CAREX008	F01.166	(TC)9	CAGTATGGTGGTGAGAGCGA	CACAGACCGAACCTAACAACAA
CAREX010	F01.180	(TC)7	GCTTCGTTGTCTACTAGCCCC	GACCAATCCAGCTGAGAAGC
CAREX012	F01.240	(CT)15	CCACACAGCTTATTGCTTGC	TGATAGGTGGGTTTCTTGCC
CAREX015	F01.339	(AG)10	TGATTTTTCCAATGCGTGAA	TGCCAGTTGAATCTCAGTGC
CAREX016	F01.3449	(AG)7	CCCCTTCAATTCAATGCTGT	GCAATGAGAGGGAAAATCCA
CAREX017	F01.3529	(GA)10	TGAATCATTGAAGGAGAGAGCA	GGTTGTTGCAAAGGAAGAGC
CAREX018	F01.357	(AG)7	TTTCTAACCCTTTATCGCCG	AAAATTGCCTGGAGGAGGAG
CAREX019	F01.364	(AT)6	ATCATGCGGCCAAGATAAAG	CAAGCAGGGGTGGAGAATAG
CAREX023	F01.463	(TC)6	TAGTGCTGCCAGAAAGAGCA	AAACTCAACCCGAAAAGGCT
CAREX024	F01.501	(CT)8	GTCCCCAAAACCTCTGTAGC	TGCTTGTTGTTCGCTTCATC
CAREX025	F01.5079	(AG)10	TGCATGCAGCTGGAATAGAG	CTCCAAATCCGAACTATCCG
CAREX026	F01.5099	(CT)6	AGCCTCTCTCTCTCTCCGCT	GCAAAAATGCCTGAGTGGAT
CAREX030	F01.608	(CT)14	CGCCTCTTCATCGATCTTTC	GCCCCAATAATGGAGAGGAT
CAREX031	F01.612	(CT)9	CCCAATCTCCAAAGAGCAAA	AGAACGAGACCTGGAGCTGA
CAREX037	F01.735	(AT)6	GCAGGGTTGTTGAAGGTTTG	TTGTGGATGCAAAACAGCAT
CAREX040	F01.789	(TG)17	TGCTGTTCTCATGGCTTCTG	CCTTTCATTTTGATGAGGCAA
CAREX041	F01.792	(AG)7	CAAAAAGGAAGCGAAAGGAA	TGAGAGAGGAGATCGGAGGA
CAREX042	F01.809	(TA)8	ACAAAAGAGCTCGCTGGAAA	TCTGATTGCTGCTCAACTATCTC
CAREX048	F01.1070	(GCT)5	CTTCATTTCCGCCTCTCTTG	GCAATCATTATGCAATGCCA
CAREX044	F01.854	(CT)6	ACACAGGGACAAGCCGATAG	CAACAAGCACAACAATAACCA
CAREX045	F01.855	(CT)6	CCAAAAGATATCATCATCTCCGA	TGAGCAGCGATCTCTTTGAA
CAREX046	F01.930	(AT)6	TCTTTTTGCCAAGATGGTGA	GTGCCAAGCATCAATCAGAA
CAREX050	F01.151	(CTC)6	TCATTAGCTGGTCGCTTCCT	CATGCCCATTGTTCTTGATG
CAREX053	F01.3450	(CTG)5	ACCCAGTGATCGTACTTCGG	AGATTCAATTTCCACCGTCG
CAREX054	F01.3506	(ACC)5	TCAACCCGCTACACCTAACC	ACACGCTCCAGGTCAGAGAT
CAREX055	F01.3514	(AAC)5	CCACACCTCCTACTCCTCCA	CTCCCCGTTGAAGTTGTTGT
CAREX060	F01.5072	(ATT)5	TACGCCATTGTCAACGAAAA	GCACGAGACACCTGAACACA
CAREX062	F01.5103	(CAG)5	AGGCACCACAAGATCCAAAC	GCTCCCATCCATACAGCTTC
CAREX064	F01.511	(CAC)9	TCCTCAGGTAGCGAAAGCAG	TACCTTAATTGCGGAATCGG
CAREX069	F01.52166	(ATA)5	GTACCTGCCCTGGATTCTGA	GGCGCTTTTACCAAATCAAA
CAREX070	F01.592	(ATT)6	ATTCATCAGGCAATTCTGGC	GGCTAAACCAACTCCTGCAC
CAREX072	F01.675	(GAT)5	CTGATCTCAAAAGGCGAAGG	CACGTAGGGATCACCCAATC
CAREX073	F01.719	(AGA)5	TAAAATGAAGGGCGAGGATG	CACTCCGTGATGATGTGGTC
CAREX076	F01.785	(GAT)8	TGAAGAAGGGCCTGGTACTG	AACCACCAGATCCCACAGTC
CAREX083	F01.1429	(AGA)5	GGGAATCACAGACAAAGGGA	ATACTGGCAGAACCAATGGC
CAREX086	F01.11991	(CTT)6	TACGGTACAGGCGTCTCTCA	CAGAAGCAACGCAACACATT
CAREX094	F01.9172	(GAAT)5	ATGGTTTCTGATTTCCTGCG	GGGGTCAAATGTAGTTGCAGA
CAREX095	F01.9272	(TAAT)8	CCTTCAAAAGAGAACCGAGC	TGTCGCCTTTGTGAGCATAG
CAREX096	F01.17075	(TCTCT)5	GACTCCGACTCCAGTTGAGC	TCGAGGAGCTGTCCTTGAAT
CAREX097	F01.21485	(CCTCT)5	CCCATCATCGATCAATCACA	GGAACAACGATCGGAAAGAA

### Genetic diversity analysis

A binary qualitative data matrix was constructed and analyzed using POPGENE Version 1.3.2. Genetic diversity of different materials was determined by calculating the percentage of polymorphic bands (PPB), the effective number of alleles (Ne), observed number of alleles (Na), Nei’s gene diversity (H), and Shannon’s information index (I). The polymorphic information (PIC) of a band was calculated by the following formula: $\mathrm{PIC}=1-\sum \limits_j^i{P_{ij}}^2$ (P*i* and P*j* are the frequencies of the *i*th and *j*th alleles at one locus). We used the software NTSYS Version 2.1 [49] to construct a cluster analysis description of selected group Q pattern based on Nei’s genetic distances [50]. Afterwards, the unweighted pairwise method of arithmetic mean is used to analyze the parameters.

### Cluster analysis and AMOVA analysis of 79 accessions

The similarity of *Carex* was evaluated using NTSYS Version 2.1. According to the similarity matrix of SSR data set, the UPGMA clustering method was used to construct the dendrogram [51]. Using PowerMarker version 3.25 and MEGA 5, the unweighted phylogenetic tree was constructed based on the Dice dissimilarity matrix between 79 individuals [52, 53]. Based on the Bootstrap function of FreeTree program, the robustness of phylogenetic trees was evaluated through 1000 repeated bootstrap analyses [54]. Principal coordinate analysis (PCoA) was performed according to the anastomotic differences between binary genotypic profiles using the GenAlEx 6.5 program based on the pairwise distance matrix [55]. Both distance and covariance were standardized.

The tratified genetic variation between and within geographic groups was analyzed using Analysis of Molecular Variance (AMOVA) [56]. F statistic was used to analyze the genetic differentiation between populations. Both analyses were performed using the GenAlEx 6.5 software [55].

## Supplementary Information

**Additional file 1: Table S1.** The information of 11 *Carex* materials to select primers.

**Additional file 2: Table S2.** The Nei’s genetic distance between *Carex* species.

**Additional file 3: Table S3.** The fingerprint of 79 *Carex* materials.

## Data Availability

The Illumina NGS reads generated in this study has been submitted to the BioProject database of National Center for Biotechnology Information (PRJNA488506).

## Abbreviations

AFLP: Amplified fragment length polymorphism
AMOVA: Analysis of molecular variance
GO: Gene ontology
H: The gene diversity index
I: The Shannon information index
ISSR: Inter-simple sequence repeat
KEGG: Kyoto encyclopedia of genes and genomes
KOG: EuKaryotic ortholog groups
Na: The average number of observed alleles
Ne: The effective alleles
NJ: Neighbor-joining
NR: NCBI non-redundant protein databases
PCoA: Principal coordinate analysis
PCR: Polymerase chain reaction
PIC: The polymorphic information content
RAPD: Random amplified polymorphic
RFLP: Restriction fragment length polymorphism
SRAP: Sequence related amplified polymorphism
SSR: Simple sequence repeat
